# Study of antimicrobial activity of Unani poly herbal toothpaste “*Sunoon Zard*”

**DOI:** 10.1016/j.heliyon.2021.e06249

**Published:** 2021-02-24

**Authors:** Ayesha Parveen, Qazi Zaid Ahmad, Mohammad Rashid, Aziz ur Rahman, Sumbul Rehman

**Affiliations:** a Department of Saidla (Pharmacy), Faculty of Unani Medicine, India; b Department of Ilmul Advia (Pharmacology), Faculty of Unani Medicine, India

**Keywords:** *Sanoon Zard*, Toothpaste, Antibacterial activity

## Abstract

**Objective:**

The present study was envisioned to develop *Sunoon Zard* a traditional Unani toothpowder into toothpaste form along with its physicochemical standardization and evaluation of anti microbial activity against oral pathogens by *in vitro* study.

**Materials and methods:**

Herbal extracts based powder was redesigned to toothpaste as per the Pharmacopoeial guidelines and its pharmaceutical evaluation was conceded as per the Indian Government Tooth Paste Specifications. In vitro study was done to evaluate the antibacterial activity by using agar well diffusion method against dental pathogens. Zone of Inhibition was taken as the end parameter against the test pathogens after appropriate incubation period. It was compared with Dimethyl sulphoxide (DMSO) used as solvent (0.01%) as Negative control whereas Ciprofloxacin 5μg/disk (standard antibiotic for gram positive) and Gentamicin 10μg/disk (standard antibiotic for gram negative) were used as Positive control. All the experiment was done as per the Clinical and Laboratory Standards Institute (CLSI) Guidelines in triplicates.

**Results:**

*Sunoon Zard* was developed into toothpaste form and its physicochemical values were found to in consonance with the optimum values as mentioned in Bureau of Indian Standard. *In vitro* study of the *Sunoon Zard* toothpaste was found to be effective against various dental pathogens with specific sensitivity with good zone of inhibition towards gram negative bacterial strains viz. *P.aeruginosa* and *K.pneuomoniae* while among gram positive a significant inhibition was found against *C.xerosis* and *S.viridans*.

**Conclusion:**

The developed toothpaste from classical Unani herbal tooth powder will provide the better patient compliance. Moreover its scientific screening which exhibited potential antibacterial activity in controlling pathogenic oral microflora compared to the standard drugs also revalidated the claim of Unani Physicians that the *Sunoon Zard* is quite effective in various oro-dental disorders.

## 1 Introduction

Nowadays oro-dental disorders like tooth decay, dental caries, gingivitis, and periodontitis have increased prevalence among all age groups because of change in various factors as life style, dietary habits, bad oral hygiene etc. These conditions adversely affect the oral cavity and aesthetic appearance [1, 2].

In recent years, people became more conscious about the side-effects of fluoride and totally chemical based toothpastes, therefore a growing resurgence towards the demand of herbal toothpaste of being a safe and natural product. In sight of above some novel phototherapeutic compounds have been developed and investigated for its efficacy [3, 4]. Herbal toothpastes are reported to have a good antibacterial activity and are safer as compared to fluoride - and triclosan-containing toothpastes [5].

“*Sunoon Zard*” (SZ) a Unani herbal product is generally used and prescribed by the Unani Physician in various oro-dental disorders and acclaimed to therapeutically manage the inflammatory and infectious condition of oral cavity [6]. But till now, there is no scientific study carried out to revalidate this claim of Unani scholars of using SZ in oro-dental ailments. However, the ingredients of SZ has been reported to possess significant antibacterial activity individually [6, 7]. Therefore, present study is designed to scientifically revalidate the claims of Unani literature to use SZ for infective disorders of oral cavity, along with this an approach has been made to redesign the powder form of SZ to toothpaste for easy and convenient use.

## 2 Materials and methods

### 2.1 Composition of redesigned dosage form: powder to toothpaste

The composition of *Sunoon Zard* was used as mentioned in Hamdard Pharmacopoeia and Ifada-e-kabeer [8, 9] as shown in Table (1). It has been redesigned to toothpaste form as per Guidelines.

**Table 1:** Ingredients of *Sunoon Zard*

S.No	Ingredients	Scientific Name	Quantity
**1**	Post-e-Anaar	*Punica granatum*Linn.	50 gm
**2**	Sumaq	*Rhus coriara* Linn.	50 gm
**3**	Gul-e-Anaar	*Punica granatum*Linn.	50 gm
**4**	Mazoo-e-sabz	*Quercus infectoria* Oliv.	50 gm
**5**	Haldi	*Curcuma longa* Linn.	50 gm
**6**	Phitkari Biryan	Burnt Potassium Aluminum Phosphate	50 gm

### 2.2 Collection, processing and authentication of crude drugs -SZ

The ingredients of *Sunoon Zard* were procured from the drug store of Dawakhana (Pharmacy) Ajmal Khan Tibbiya College, Aligarh Muslim University, Aligarh (INDIA). The raw drugs were identified and authenticated by Botanist Prof. S.H. Afaq Dept. of Ilmul Advia, AMU, and specimen of every ingredient are submitted in Department of Saidla for future reference [Voucher no. 326/2017/SFUM]. The crude drugs were cleared from any foreign matter and dried in oven at 40 °C. Coarse powder of crude drugs was made by grinding them up to the size of 0.1 micron and it was confirmed by passing them through 60 # mesh size filter. Other chemicals used to redesign powder to toothpaste were of analytical grade, and were obtained from Hi-media India Ltd and Merck India Ltd.

### 2.3 Preparation of the herbal extract

100 g of each of the powdered ingredient of SZ was exhaustively extracted separately in 800 mL of 95% v/v ethanol using Soxhlet extractor for six consecutive hours. The filtered extracts so obtained were dried with rotary evaporator. The percentage recovery of extract was calculated as: (mass of extract x 100%)/mass of powdered plant. The concentrated extracts (8.34 gm) was kept separately in a clean and dried glass container.

Toothpaste was prepared by taking 0.7% extract of all the plant drugs separately except mineral drug (Potassium Aluminum Phosphate)*,* which was mixed separately (see Table 2).

**Table 2:** Composition of toothpaste.

S. No	Ingredients	Qty. Used (%w/w)	Property
1	Extract + Potassium Aluminum Phosphate	(0.6 gm + 1 gm)	Active Ingredients
2	Calcium carbonate	25 gm	Abrasive
3	Sodium lauryl sulphate	1.5 gm	Surfactant
4	Sorbitol	20 gm	Humectant
5	Sodium carboxy methyl cellulose	20 gm	Binding agent
6	Sodium saccharine	0.3 gm	Sweetners
7	Methyl paraben	0.1 gm	Preservative
8	Propyl paraben	0.02 gm	Preservative
9	Titanium dioxide	0.5 gm	Opacifier
10	Clove oil	0.5 gm	Flavouring agent
11	Double Distilled water (DDW)	20 ml	Vehicle

### 2.4 Preparation of toothpaste

The toothpaste was prepared by heating liquid phase process-trituration method as per the guidelines [10].

## 3 Heated liquid-phase process

Total 0.6 g of extract of all drugs and 01 g powder of Potassium Aluminum Phosphate was weighed and mixed with Sorbitol, water and Saccharin Sodium Dehydrate with continuous stirring for 15 min at 40 °C. Calcium carbonate, carboxy methyl cellulose and preservatives (Methyl Paraben and Propyl Paraben) were mixed properly in another sterile beaker. A hot solution of humectants, water, extracts and sweetener which was prepared after proper heating and stirring was then slowly added with gentle mixing, to the powder. The resulted mass was mixed well to get thick paste. Finally the Sodium Lauryl Sulphate, peppermint oil, Titanium dioxide were added and mixed properly till a homogenous paste was formed.

### 3.1 *In vitro* antibacterial activity of toothpaste against dental pathogens

#### 3.1.1 Bacterial strains

Some clinical isolates of bacterial strains (seven gram positive and four gram negative) were selected on the basis of their clinical importance in causing oro-dental disease. The pathogenic strains were obtained from the lab of department of Microbiology, Jawaharlal Nehru Medical College & Hospital, Aligarh Muslim University, Aligarh. Bacterial strains selected for the study includes *Staphylococcus aureus (S.aurues)*, *Streptococcus mutans (S.mutans), Bacillus cereus* (*B. cereus*), *Streptococcus pyrogenes (S.pyrogenes), Streptococcus viridans (S. viridans), Staphylococcus epidermidis (S. epidermidis),* and *Corynebacterium xerosis* (*C.xerosis*) gram positive bacteria and *Escherichia coli* (*E. coli*), *Klebsiella pneuomoniae*(*K. pneuomoniae*), *Proteus vulgaris* (*P.vulgaris*) and *Pseudomonas aeruginosa* (*P*.*aeruginosa*) gram negative bacterial strains. These strains were screened for evaluation of antibacterial activities of the redesigned toothpaste form of SZ.

#### 3.1.2 Medium

Nutrient Agar No.2 (NA) (M 1269S-500G, Hi-media Labs Pvt. Ltd, Bombay, India) was used as the solid media namely for preparing nutrient plates, while Nutrient Broth (NB) (M002-500G, Hi-media Labs Pvt. Ltd, Bombay, India) was used for the preparation of liquid culture media.

### 3.2 In-vitro antibacterial activity

The antibacterial activity of the toothpaste form was evaluated by agar well diffusion method [11]. All the microbial cultures were adjusted to 0.5 McFarland standards, which is visually comparable to a microbial suspension of 1.5 Х 10^8^ cfu/ml. Autoclaved agar media (20 ml) was poured into each petri plate, followed by the swabbing of bacterial colony from the inoculums of the test microorganisms on prepared media plates, which were then incubated for 15 min at 37 °C to allow proper adsorption and active growth of the pathogen. It was followed by pouring of the test sample (100 μl reconstituted in the dimethyl sulphoxide-DMSO 0.1%) into the wells of 6 mm diameter (bored by sterile core-borer) of the seeded agar plates. Subsequently dimethyl sulphoxide-DMSO 0.1%- (the solvent used to reconstitute the test sample) was also poured to assess its activity, if any (Negative Control) and standard antibiotic disc- Ciprofloxacin 5 μg/disk (for gram positive) and Gentamicin 10 μg/disk (for gram negative) (Positive control) was placed in the same plate. All the plates were incubated at 37 °C for 24 h.

After incubation the antimicrobial activity of toothpaste form was evaluated by measuring the zone of growth inhibition against the test microorganisms with Antibiotic Zone Scale (PW297, Hi-media Labs Pvt. Ltd., Mumbai, India), which was holded over the back of the inverted plate. The plate was held a few inches above a black, nonreflecting background and illuminated with reflected light. The experiments were performed in triplicates.

## 4 Observations and results

### 4.1 Physico-chemical standards of toothpaste

. (see Tables 3 and 4)

**Table 3:** Physico-chemical appearance of toothpaste.

1.	Colour	Dull yellow
2.	Taste	Sweet
3.	Odour	Peppermint
4.	Homogeneity	Homogenous
5.	Smoothness	Smooth
6.	Elegancy	Aesthetic appearance
7.	Spread ability	6.2 ± 0.115 (cm)
8.	Tube Extrudibility	62.67 ± 1.45 (%)
9.	Fineness of Toothpaste:	62.67 ± 1.45 (% by mass)
10.	pH Value	7.6 ± 0.115
11.	Foaming Power:	7.6 ± 0.115 (ml)
12.	Dispersion Time in Water:	30 ± 1.155 (min)
13.	Volatile matters and Moisture	31.07 ± 1.569 (%)

**Table 4:** Comparison with the optimum values mentioned in Bureau of Indian Standard.

VALUES	HSA	SP	pH	FN	FF	DT
Toothpaste value	Absent	6.2	7.6	0.33	124	30
Standard value	Absent	(Max.) 8.5	5.5–10.5	(Max.) 0.5	Min 50	10–30 min

[HAS- Hard and sharp edged abrasive particles; SP- Spreadability (cm); FN- Fineness (% by mass); FF-Foam formation (ml); DT- Dispersion time in water (DT)].

### 4.2 Antibacterial susceptibility testing

**Figure 1:**
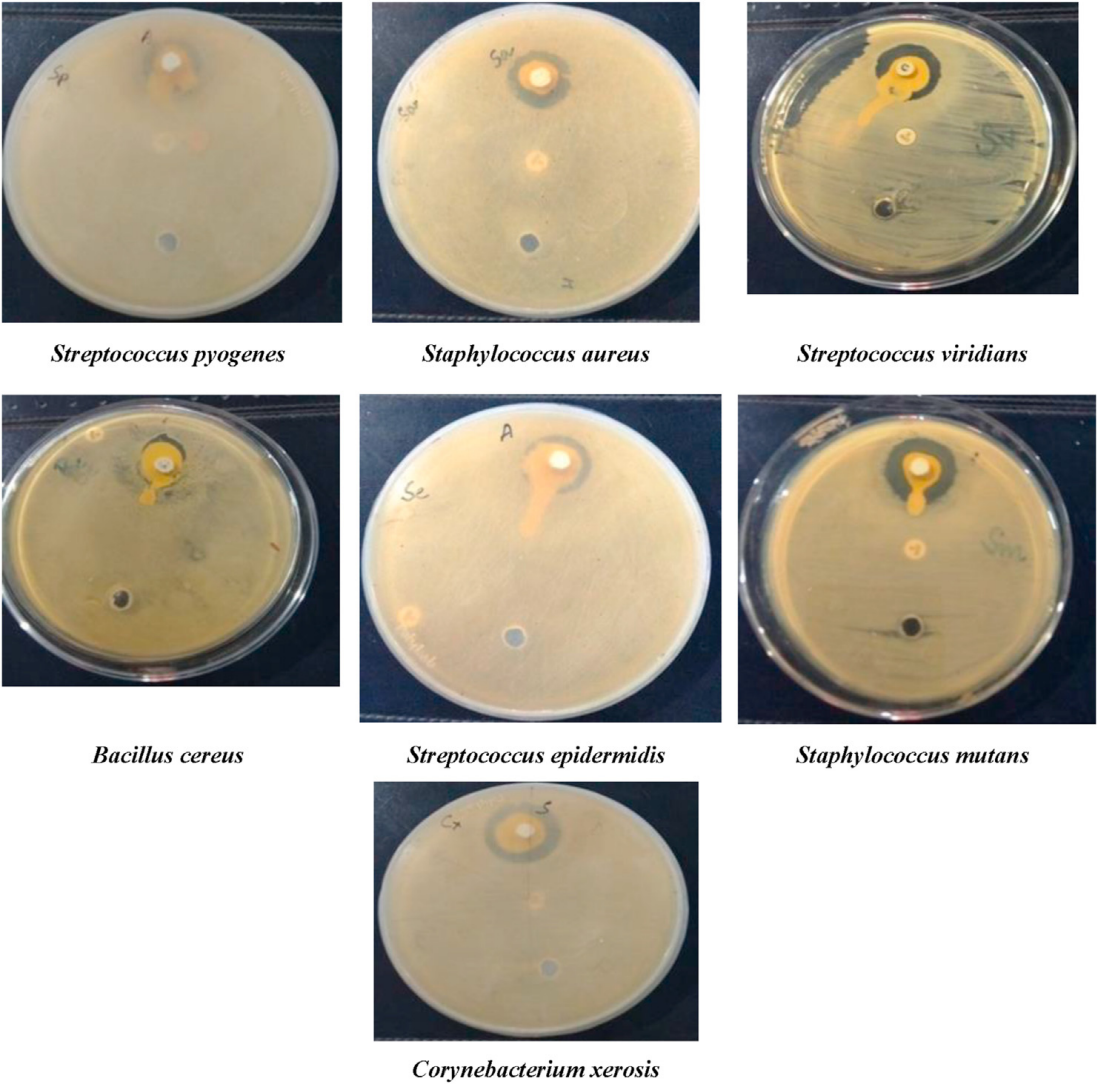
Antibacterial Activity of Test Drug sample against Gram positive bacterial strains.

**Figure 2:**
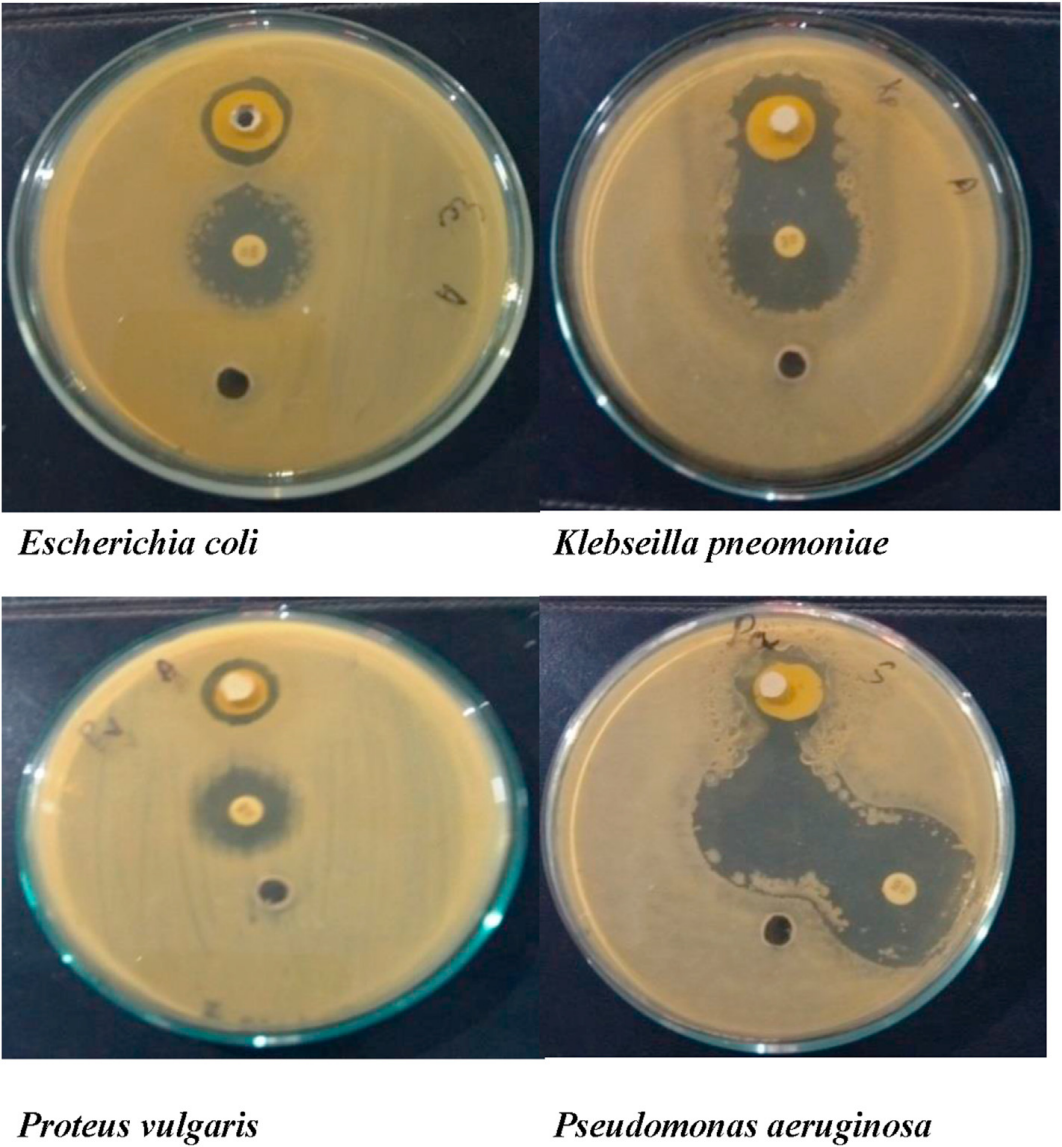
Antibacterial Activity of Test Drug sample against Gram negative bacterial strains.

**Figure 3:**
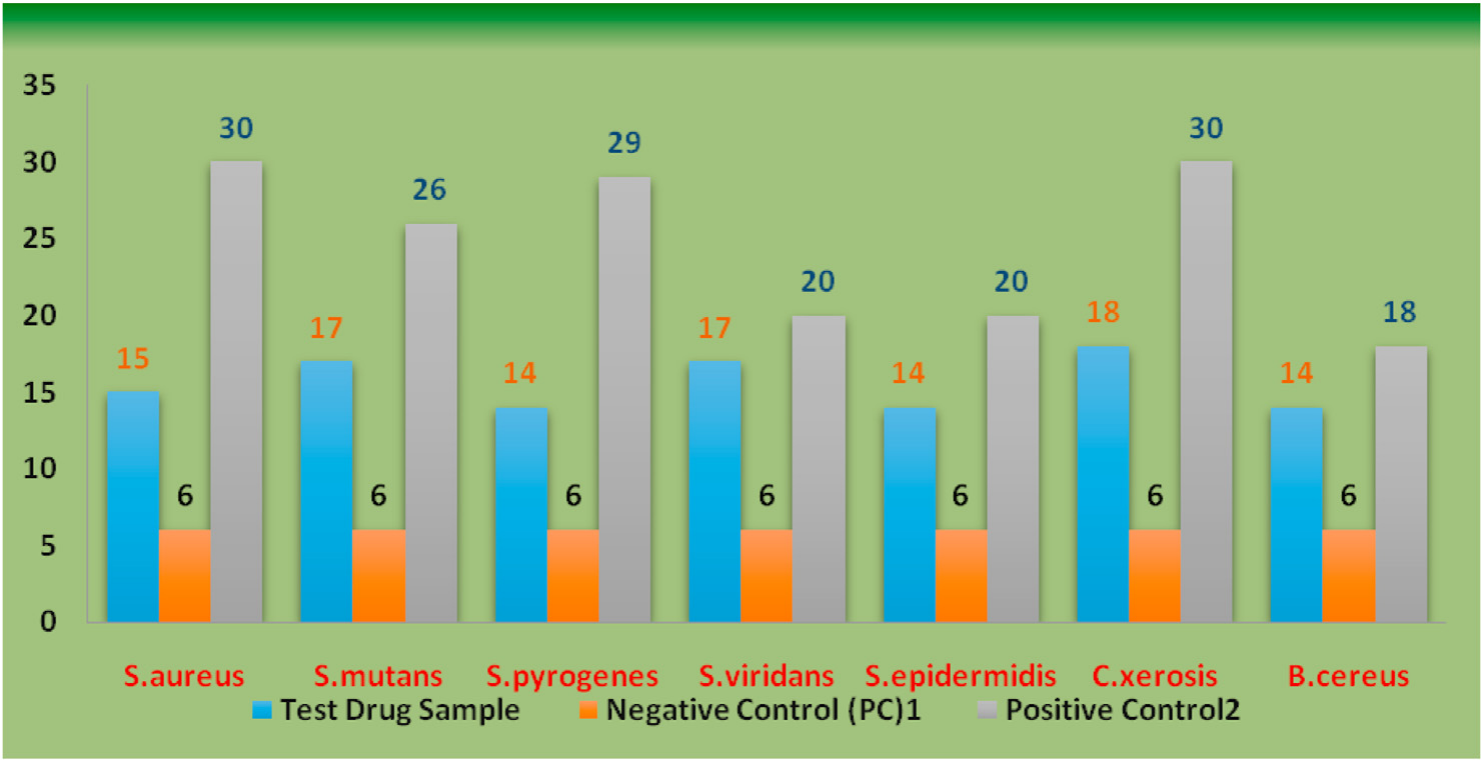
Antibacterial Activity of Test Drug sample against Gram positive bacterial strains.

**Figure 4:**
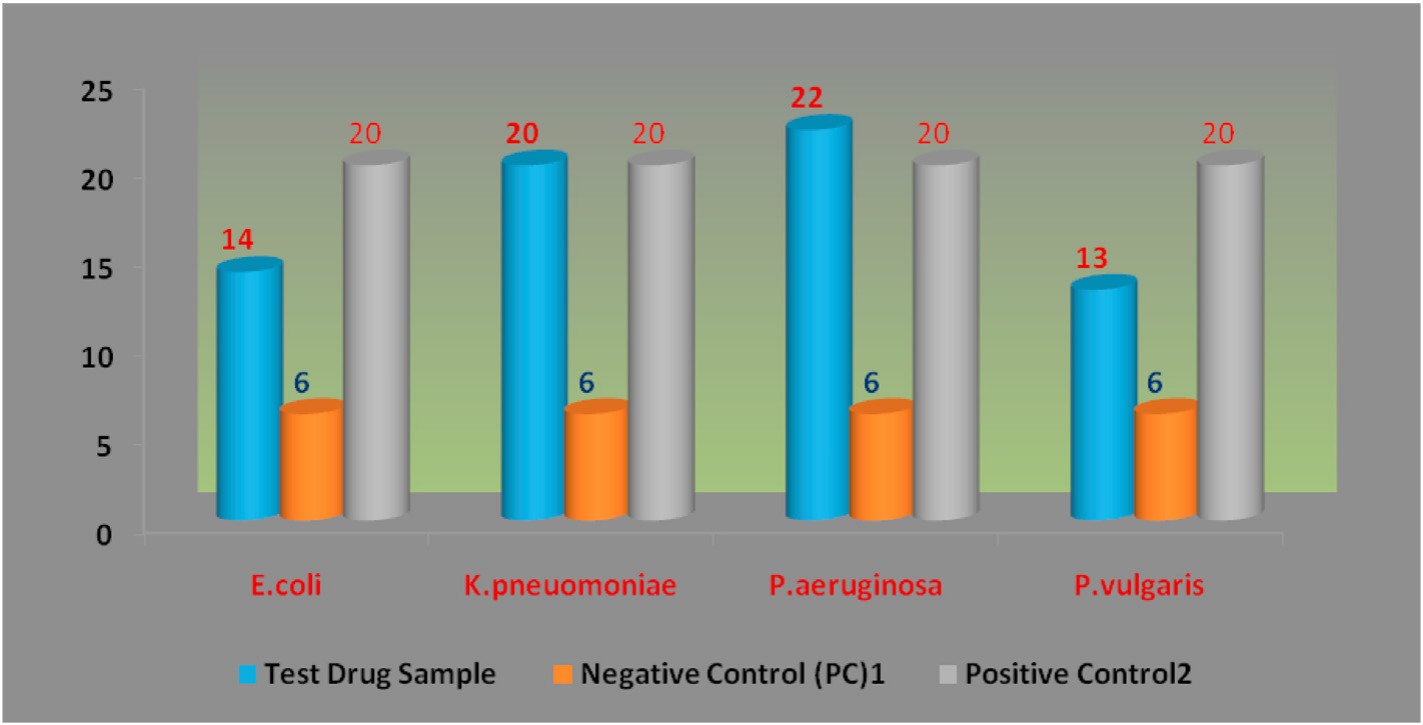
Antibacterial Activity of Test Drug sample against Gram negative bacterial strains.

Toothpaste in its prepared form was used for screening antibacterial activity using Kirby-bauer's disk diffusion method and Agar well method according to CLSI Guidelines [12, 13] against some clinical isolates of seven Gram positive and four Gram Negative bacterial strains. 50 μgm of the test drug compound (toothpaste) was used and compared with the Positive Control (Standard drug Ciprofloxacin for Gram positive and Gentamicin for Gram Negative bacteria) and Negative/Plane control i.e. DMSO [Figures 1 and 2]. The results are presented in Table No. 5 and graphically represented in Fig No. 3&4 [12,13] (see Figures 3 and 4).

**Table 5:** Antibacterial Screening: Zone of Inhibition (in mm) of the Complex against bacterial strains. Results expressed as Mean ± Standard error of Mean (Standard Deviation).

Strains	Zone of Inhibition (in mm)		
	Test Drug Sample	Negative Control^1^	Positive Control^2^
**Gram Positive Strains**			
*S.aureus*	15.33 ± 0.88(1.52)	6.66 ± 0.33(0.57)	30.66 ± 0.33(0.57)
*S.mutans*	17.66 ± 0.88(1.52)	6.33 ± 0.33(0.57)	26.66 ± 0.33(0.57)
*S.pyrogenes*	14.66 ± 0.33(0.57)	6.33 ± 0.33(0.57)	29.33 ± 0.33(0.57)
*S.viridans*	17.33 ± 0.33(0.57)	6.33 ± 0.33(0.57)	20.33 ± 0.33(0.57)
*S.epidermidis*	14.33 ± 0.33(0.57)	6.33 ± 0.33(0.57)	20.33 ± 0.33(0.57)
*C.xerosis*	18.33 ± 0.33(0.57)	6.33 ± 0.33(0.57)	30.33 ± 0.33(0.57)
*B.cereus*	14.66 ± 0.33(0.57)	6.33 ± 0.33(0.57)	18.33 ± 0.66(1.15)
**Gram Negative Strains**			
*E.coli*	14.33 ± 0.33(0.57)	6.33 ± 0.33(0.57)	20.33 ± 0.33(0.57)
*K.pneuomoniae*	20.33 ± 0.33(0.57)	6.33 ± 0.33(0.57)	20.33 ± 0.33(0.57)
*P.aeruginosa*	22.33 ± 0.33(0.57)	6.33 ± 0.33(0.57)	20.33 ± 0.33(0.57)
*P.vulgaris*	13.33 ± 0.33(0.57)	6.33 ± 0.33(0.57)	20.33 ± 0.33(0.57)

1 Dimethyl Sulphoxide.

2 Standard Drug (Ciprofloxacin for Gram positive and Gentamicin for Gram negative strains).

## 5 Discussion

Common dental diseases are tooth decay (cavities, dental caries) and gum diseases, including gingivitis and periodontitis. Among all dental caries affects approximately 60–65% in India due to sugar consumption along with many other causative factors [14].

There are number of single drugs and compound formulations which are commonly being advised by the traditional healers such as Ayurveda, Unani etc. to prevent and treat the common oro-dental disorders in children and old age patients like Miswak (*Salvadora persica*), Haldi (*Curcuma longa*), Anar (*Punica granatum*), Aqarqarha (*Anacyclus pyrethrum*), Suddab (*Rutagraveolens*), Amla (*Emblica officinalis*), Aqaqia (*Acacia nilotica*), Shahad (Honey), Lehsun (*Allium sativum*), Aspaghol (*Plantago ovata*), Babuna (*Matricaria chamomilla* Linn.), Clove (*Syzygium aromaticum*) etc. which are found to be useful in treatment of oro-facial diseases. Similarly, Unani formulations like *Sunoon Zard*, *Sunoon Mulook, Sunoon Mujalli* etc. have been shown effective anti-bacterial, anti-inflammatory and analgesic effect and are prescribed in the treatment of oro-facial diseases [5].

Antibiotics and other antimicrobial agents are effective in the prevention and treatment of dental caries, but they also cause undesirable side effects as ecologic disturbance of oral and gut flora. Further, viridians group streptococci including *S. mutans, C. albicans* and most representative human cariogenic bacteria are reported to be moderately resistant to antibiotics. All these facts have resulted in re-search and development for new antimicrobial formulations.

The Unani System of Medicine has in its treatise a number of compound formulations which are commonly being prescribed and used by the patients and found to be effective in various ailments, however these traditional dosage form couldn't be develop at modern parameter for the better compliance of the patient along with its standardization and scientific screening to validate the claim of Unani Physicians.

Natural plant products are an important source to control bacterial pathogens. Therefore, in the present study, a poly-herbal tooth powder was redesigned to toothpaste form, it was standardized as per the guidelines [15, 16, 17] and evaluated for antibacterial activity which has showed significant results.

There was a significant antibacterial activity of the test drug (depicted in table No.5) when it was compared with the standard drug Ciprofloxacin for gram positive bacterial strains. In the order of increasing efficacy it was found to be very effective against *C. xerosis* with Zone of Inhibition as up to 18 mm followed by an equivalent efficacy against *S. mutans* and *S. viridans* (ZOI 17 mm) and relatively lower efficacy but inhibition was observed for *S. aureus*, *S. epidermidis* and *B. cereus* (ZOI -14 mm). For gram negative bacterial strains the test drug was found to be more effective against *P. aeruginosa* with ZOI of 22 mm followed by *K.pneuomoniae* (ZOI-20 mm) and an inhibigtion was observed against *E. coli* (ZOI-14 mm) and *P. vulgaris* (ZOI-13 mm) compared with Gentamicin on the same media plates.

The significant activity of the polyherbal Unani tooth powder is attributed to the various natural phyto-constituents present in the natural drugs. Antibacterial activity may be related to the presence of hydrolysable tannins and polyphenolics present in the *P. granatum, C.longa, Q.infectoria*; Tannins acts on the cell wall and across the cell membrane, as they can precipitate proteins [18, 19], suppress many enzymes such as gycosyltransferases [20]. Reddy et al [21] and Naz et al [19] demonstrated that gallic acid (a tannic acid) has the highest antibacterial effect against tested sensitive strains even at low concentrations. Activity contributed by *P. granatum* can be attributed to the presence of punicalagin, gallagic acid [19, 21] and polyphenols [19, 20] present in the tested formulation which synergize the antibacterial efficacy due to their significant effect on the bacterial cell wall, they inhibit enzymes by oxidized agents, interact with proteins and disturb co-aggregation of microorganisms. Besides, methyl gallate and gallic acid, which are the main components of gallotannin from *Q.infectoria*, were found to prevent growth (bacteriostatic) of cariogenic bacteria as well as periodontal bacteria [22] tested. *C. longa* present in the formulation is reported to synergise the antibacterial efficacy due to the presence of essential oil, curcumins, curcuminoids, turmeric oil, turmerol and veleric acid [23, 24, 25].

Other mechanisms have also been proposed to justify the antibacterial activity shown by tannin like complex formation between tannin and microbial enzymes (such as cellulase) as well as their effect on membrane of microbes due to their astringent property, iron deprivation through precipitation and effect on bacterial metabolism through inhibition of oxidative phosphorylation.

In the present study, we have used the disk diffusion method for the antibacterial evaluation of Sunoon e Zard; however, the MIC method (Minimum Inhibitory Concentration) applied along with disk diffusion may be recommended in future studies.

## 6 Conclusion

The developed toothpaste from the ingredients of Sanoon Zard tooth powder will provide the better compliance to the patients. While the significant effect of the toothpaste against dental pathogens offers a scientific basis to validate the traditional knowledge. Therefore *Sanoon Zard* may be subjected for further experimental and clinical studies to explore and affirm its efficacy in various aspect including safety studies.

## Declarations

### Author contribution statement

Ayesha Parveen: Conceived and designed the experiments.

Zaid Ahmad Qazi: Conceived and designed the experiments; Wrote the paper.

Mohammad Rashid: Performed the experiments.

Sumbul Rehman: Performed the experiments; Analyzed and interpreted the data; Contributed reagents, materials, analysis tools or data.

Aziz ur Rahman: Contributed reagents, materials, analysis tools or data.

### Funding statement

This research did not receive any specific grant from funding agencies in the public, commercial, or not-for-profit sectors.

### Data availability statement

Data included in article/supplementary material/referenced in article.

### Declaration of interests statement

The authors declare no conflict of interest.

### Additional information

No additional information is available for this paper.
